# Polyelectrolyte Multilayers Composed of Polyethyleneimine-Grafted Chitosan and Polyacrylic Acid for Controlled-Drug-Delivery Applications

**DOI:** 10.3390/jfb13030131

**Published:** 2022-08-28

**Authors:** Eliz Selmin Paker, Mehmet Senel

**Affiliations:** 1 Department of Chemistry, Istanbul University, Istanbul 34521, Turkey; 2 Department of Biochemistry, Faculty of Pharmacy, Biruni University, Topkapi, Istanbul 34010, Turkey

**Keywords:** layer-by-layer film, chitosan, polyethylene imine, polyacrylic acid, drug delivery

## Abstract

In this work, polyethyleneimine (PEI)-grafted chitosan (Chi-g-PEI) was prepared for the fabrication of layer-by-layer (LBL) films for use in sustained-drug-delivery applications. Chi-g-PEI and polyacrylic acid (PAA) multilayer films were formed using the LBL technique. Methylene blue (MB) was used as a model drug for the investigation of loading and release capabilities of the LBL films. Characterizations of the synthesized copolymer were performed using Fourier-transform infrared spectroscopy (FTIR), Nuclear magnetic resonance spectroscopy (NMR), Thermogravimetric analysis (TGA), and X-ray Powder Diffraction (XRD) techniques, and the thickness of the LBL films was measured using Atomic force microscopy (AFM). The drug-loading and -release behaviors of the LBL films were assessed using a UV–visible spectrophotometer. The results showed that the loading capacity and release rate of MB were affected by ionic strength and pH. In addition, it was demonstrated that PEI-grafted chitosan is a good candidate for the assembling of LBL films for drug-delivery applications.

## 1. Introduction

The delivery of drugs is a vital part of medicine and healthcare services [1]. Drug-delivery systems can help to control the pharmaceutical effect of drugs. These systems can affect the pharmacokinetics of a drug, the drug release rate, the site and duration of drug action, and afterwards the side-effect profile [2]. Ideally, a drug-delivery system could deliver a certain amount of a drug to the site of action at a certain rate in order to maximize the desired therapeutic response [3,4]. Layer-by-layer deposition has special interesting advantages in the field of the delivery of biologicals, such as nano-scale film architecture allowing the fine control of the loading and release of drugs to be performed, the ability to coat difficult geometries, and ease of fabrication. The LBL self-assembly method is an extremely versatile, reproducible, and efficient way for modifying surfaces as well as fabricating polymeric multilayer thin films with proposed structures, thicknesses, compositions, properties, and functions over any type of substrate [5].

The formation of multilayer thin films through the sequential adsorption of positively and negatively charged polymers on solid surfaces through electrostatic attraction in a layer-by-layer manner was first proposed by Iler in 1966 [5]. Since then, this method has received large interest, and applications have been expanded. LBL multilayer films can be formed with a variety of deposition methods, such as dip coating, spin coating, and spraying [6,7]. Among these, the dip-coating method is extensively used to build LBL assemblies because of its ability of coating substrates with complex geometries. This technique is applied in completely aqueous solutions, and it is very convenient method for the formation of homogeneous multilayer films. In addition, changing the applied external stimuli, such as ionic strength, pH, temperature, electrical field, light, and mechanical stress, or adding specific biological moieties, such as proteins or ionic surfactants, can modify the multilayers or even induce their desorption behavior, thus inducing other intermolecular interactions of great importance [8]. One of the most abundant polysaccharides in the nature is chitosan (Cts). Due to its nontoxicity, biocompatibility, and slow biodegradability characteristics, Cts has gained attention in recent decades and plays major roles in many disciplines, such as chemistry, analytical chemistry, catalysis, biomedicine, and nanotechnology [9,10,11,12,13,14]. Cts may form poly-ionic complexes with carboxylic acid functional molecules because of the abundance of its amine groups.

It has limited applications due to low solubility in neutral and basic media. Grafting chitosan can improve its solubility and widen its applications, and because of that, it is the most attractive way to modify it [15]. One of the most popular polyelectrolytes for LBL multilayer film fabrication is poly(acrylic acid) (PAA). Due to its weak polyelectrolyte behavior, films formed with PAA show pH-triggered drug release [16]. In addition, polyethyleneimine (PEI) branching has been attracting interest due to its common use in the pharmaceutical industry and in the stabilizing of multilayer films [17,18].

In this work, we designed PEI-grafted chitosan (Chi-g-PEI) using an imine reaction between low-molecular-weight PEI and periodate-oxidized chitosan to be able to use it for LBL multilayer film fabrication and for enhancing the loading capacity of chitosan. Modified chitosan was used to prepare LBL films with polyacrylic acid, and methylene blue was chosen as the model drug molecule to visualize the drug-loading and -release behaviors of the films. LBL Chi-g-PEI films can be used in the form of films on flat surfaces, nanoparticles, and nanocapsules for drug-delivery applications, such as nasal, ocular, or gastrointestinal applications [19,20]. Therefore, the novelty of this work lies in the development of layer-by-layer self-assembled monolayers on a surface for potential controlled-drug-delivery applications. To our knowledge, no other paper has described such monolayer formation using PEI-grafted chitosan as a delivery system that provides the slow release of drugs due to the swelling and dissolution of the polymer matrix and an increased rate due to its pH-responsive behavior.

## 2. Materials and Methods

### 2.1. Materials

Chitosan (MW 50–190 kDa; deacetylation degree: 75–85%), polyethylene imine (MW 25 kDa), potassium periodate, and sodium chloride were obtained from Sigma Aldrich (Dortmund, Germany). Methylene blue was obtained from Carlo Erba Reagent (Val-de-Reuil, France). Polyacrylic acid (PAA) (MW 150–170 kDa) was synthesized using radical polymerization as described in our previous study [21]. 

### 2.2. Synthesis of PEI-Grafted Chitosan

**Figure 1:**
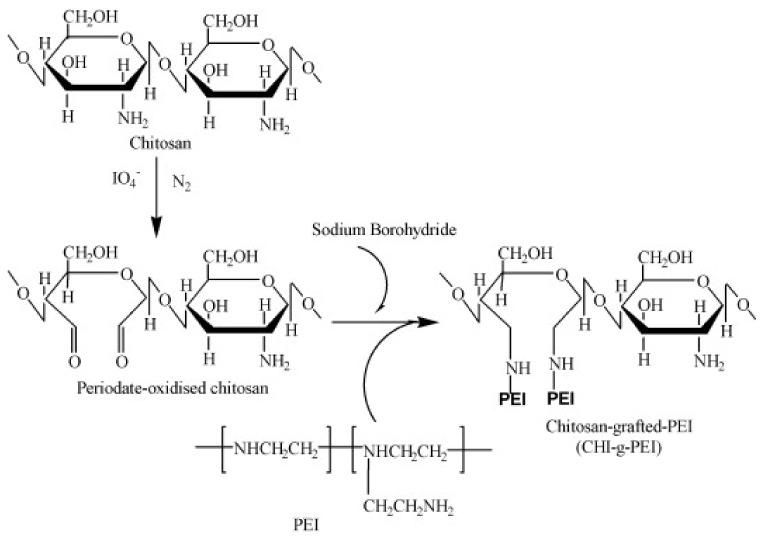
Reaction scheme for preparation of Chi-g-PEI.

PEI-grafted chitosan (Chi-g-PEI) was prepared according to the literature in two steps (Figure 1) [22]. Firstly, the Cts-oxidation step was performed via the treatment of LMW Cts (0.1 M) with KIO_4_ (0.01 M) in sodium acetate buffer (pH 4.5). The solutions were mixed after each solution was adjusted to 4 °C; the reaction was run for 48 h at room temperature and stopped by adding ethylene glycol (10% *v*/*v*). The final oxidized Cts was purified following a two-step dialysis procedure (Spectra/Por® membrane: MWCO = 3500) against NaCl (0.2 M; pH 4.5) and with deionized water (pH 4.5). Secondly, PEI (20 mmol) was allowed to react with periodate-oxidized Cts solution (10 mmol) by stirring for 2 days at 4 °C. Then, the solution was treated with sodium borohydride (2 g NaBH_4_/g chitosan) and dialyzed against NaCl (0.2 M; pH 4.5) (MWCO = 12–14 K) and against deionized water (pH 4.5) at 4 °C [21]. After the dialysis procedure, the final product (Chi-g-PEI) was lyophilized (yield: 86%).

### 2.3. Preparation of Multilayer (LBL) Films

**Figure 2:**
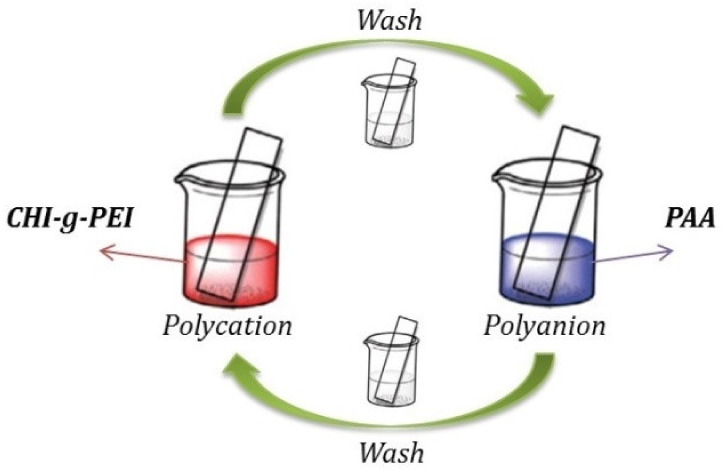
Schematization of LBL film formation with Chi-g-PEI and PAA.

To be able to remove the residues of organic impurities from the glass slides, they were pre-treated with piranha solution (3:1 *v*/*v* mixture of 98% H_2_SO_4_ and 30% H_2_O_2_) for 60 min and then rinsed with deionized water and dried. The multilayer films of Chi-g-PEI and PAA were prepared according to the following procedure (illustrated in Figure 2): The treated slides were firstly immersed in Chi-g-PEI solution (1 mg/mL; pH 3.2) for 2 min, followed by rinsing in two separate beakers of deionized water for 1 min, and then dried with air flow. Then, the Chi-g-PEI-deposited slide was dipped in PAA solution (1 mg/mL; pH 3.2) for 2 min, followed by rinsing in two separate beakers of deionized water for 1 min, and then dried with air flow. The process was repeated ten times until a Chi-g-PEI/PAA film with the desired layers was deposited.

### 2.4. Methylene Blue Loading and Release

LBL-coated glass slides were dipped into 0.3 mg/mL methylene blue solutions with different pH values and NaCl concentrations for 20 min. The films were taken out after 20 min and then rinsed with deionized water and dried under air flow. During this process, 1000 μL of the sample was collected from the outer phase every 2 min. The loading process of MB into (Chi-g-PEI/PAA)_10_ films was monitored using UV–Vis spectroscopy. 

The methylene blue-loaded LBL-film-coated glass slides were dipped in NaCl solutions at different NaCl concentrations and aqueous solutions with different pH values for release studies. A volume of 1000 μL of the sample was withdrawn from the outer phase; then, 1000 μL of solution was added to the outer phase during mechanical stirring. This process was repeated until the concentration value was constant.

### 2.5. Instrumentation

FTIR-ATR measurements (4000–400 cm^−1^) were recorded with a Bruker spectrometer. A total of 64 spectra in the range of 4000–400 cm^−1^ were recorded with a resolution of 1 cm^−1^ and then averaged. ^1^H NMR spectra were recorded at room temperature in CDCl_3_ (containing 0.03% tetramethylsilane as internal reference; δ = 0.00 ppm) with a Bruker 400 MHz spectrometer at 300 or 400 MHz. X-ray powder diffraction (XRD) analyses were performed on a Rigaku SmartLab diffractometer (Rigaku, Tokyo, Japan) operated at 40 kV and 35 mA using Cu Kα radiation. The thermal stability was determined with a thermogravimetric analysis (TGA; Perkin Elmer Instruments model; STA 6000, Waltham, MA, USA). The TGA thermograms were recorded using 5 mg of powder sample at a heating rate of 10 C/min in the temperature range of 30–800 °C in nitrogen atmosphere. Atomic force microscopy (AFM) was performed using Asylum Research MFP-3D-AS operated in tapping mode. To measure the thickness, the multilayered films were scratched using a scalpel, and the heights of the grooves were determined using AFM. A UV–visible analysis was carried out using a Shimadzu UV-2600 UV–Vis spectrometer.

## 3. Results and Discussion

### 3.1. Synthesis and Characterizations

PEI-grafted chitosan was synthesized by following the reaction illustrated in Figure 1. PEI was allowed to react with reduced chitosan according to a previous study [22]. Before grafting, Chi was selectively oxidized with periodate. Periodate oxidation can selectively oxidize vicinal diols and cleave the C-C carbon bonds to introduce aldehyde groups, which expands the promising application of carbohydrate polymers [23,24]. The reaction conditions (temperature, amount of PEI) were adjusted carefully during the grafting of chitosan in order to avoid gelation. The degree of grafting was determined with the integral values from the proton peaks of PEI and was found to be 8.3 mol%.

**Figure 3:**
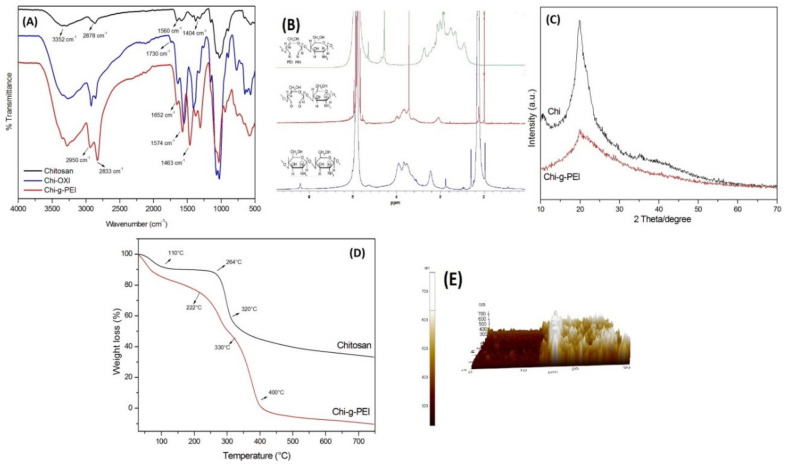
(**A**) FTIR and (**B**) ^1^H NMR spectra of chitosan, Chi-OXI, and Chi-g-PEI. (**C**) XRD diffractograms of chitosan and Chi-g-PEI. (**D**) TGA analysis of chitosan and Chi-g-PEI. (**E**) AFM image of (Chi-g-PEI/PAA)_10_ multilayer film on glass slide.

Figure 3A shows the FTIR spectra of Chi, Chi-OXI, and Chi-g-PEI. The FTIR spectra of Chi had characteristic peaks at 3352 and 2878 cm^−1^, which were attributed to –OH and –CH_3_ groups. The typical -NH bending vibration and –OH vibrations were seen at 1560 and 1404 cm^−1^, respectively. Furthermore, the bands at 1320 and 1077 cm^−1^ were attributed to the stretching of C-O-N and C-O groups. The bands at 1154 and 897 cm^−1^ corresponded to the stretching of C-O-N and C-O functional groups [25]. The stretching of the -C = O group was seen at 1740–1720 cm^−1^. The second spectrum of oxidized chitosan (Chi-OXI) showed an absorption band at 1730 cm^−1^, which was attributed to the formed –C = O aldehyde groups. The third-spectrum band belonged to PEI-grafted chitosan. The main functional groups in the ethylene imine units of PEI are –CH_2_, −NH, and –NH_2_. The spectrum of PEI showed a peak at 1463 cm^−1^, which was attributed to the –CH_2_ group. The band at 1574 cm^−1^ corresponded to the vibration of the –NH group and the stretching vibration of the C-N groups of PEI [26]. The bands seen at 2833 and 2950 cm^−1^ were due to the asymmetric and symmetric stretching of the –CH_2_ groups [17]. Moreover, the peak observed at 1652 cm^−1^ corresponded to the –NH_2_ groups.

The modification of chitosan with PEI was checked using ^1^H NMR spectroscopy (Figure 3B). After the oxidation of chitosan, the decrease in the peak of 2—Carbon of Chi was attributed to the cleavage of the carbon–carbon bond by the periodate ion and the formation of dialdehyde [22,27,28]. As seen in Figure 3B, the peaks at 3.3–2.5 ppm were attributed to the grafting of chitosan with PEI (-NHCH_2_CH_2_-).

The X-ray diffraction patterns of Chi and Chi-g-PEI are shown in Figure 3C. Chitosan had characteristic peaks 2*θ* = 10 and 2*θ* = 20, which were assigned to crystal forms I and II, respectively [29]. After the grafting with PEI, the intensity of the peaks at 10° and 20° was significantly decreased due to the destruction of the intermolecular hydrogen bonds between the amine groups and the hydroxyl groups of chitosan [30].

Figure 3D shows the thermograms of Chi and Chi-g-PEI under heating in an inert atmosphere. The first stages of the weight loss of Chi and Chi-g-PEI belonged to bound water. Chitosan showed slow weight loss starting from 120 to 250 °C due to the decomposition of the polymer with low molecular weight, followed by more obvious loss of weight starting from 260 to 320 °C, which could be attributed to a complex process including the dehydration of the saccharide rings and the depolymerization and decomposition of the acetylated and deacetylated units of the polymer [31]. A fast weight loss appeared in the TG curve of PEI grafted Chi decomposing from 220 to 330 °C After grafting with PEI, the thermal stability of Chi was decreased when compared with original Chi. That was due to the disruption of the crystalline structure of Chi and especially to the loss of hydrogen bonding.

The thickness of LBL films was determined using AFM techniques. Multilayer LBL films of Chi-g-PEI and PAA were prepared on glass slides forming up to 10 layers, and the AFM images of the layers were measured. Figure 3E shows the incision side of the film, and the height of the incision was equal to the thickness of the coated film. The film thickness was ~0.2 µm as found from the AFM image.

### 3.2. Loading of Methylene Blue

**Figure 4:**
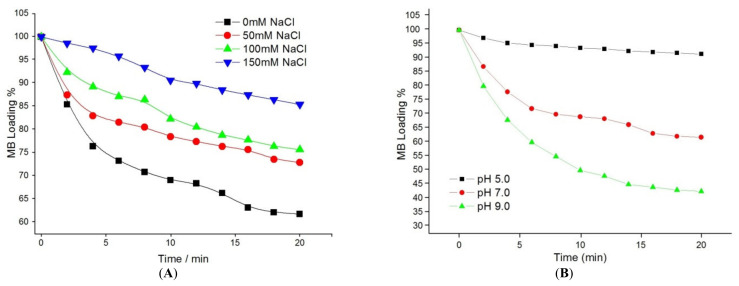
(**A**) Effects of ionic strength on MB loading into (Chi-g-PEI/PAA)_10_ multilayer films (at pH 7.0, containing 0, 50, 100, and 150 mM NaCl). (**B**) pH effect on loading of MB into (Chi-g-PEI/PAA)_10_ multilayer films (solutions containing 0 mM NaCl at pH 5.0, 7.0, and 9.0).

During the loading process, the maximum absorption wavelength of MB, 668 nm, was used in subsequent experiments to calculate the amount of loaded MB on the (Chi-g-PEI/PAA)_10_ multilayer films assembled on slides under various conditions. Figure 4 shows the effects of ionic strength and pH value on MB loading into (Chi-g-PEI/PAA)_10_ films. Figure 4A shows the immersion of the (Chi-g-PEI/PAA)_10_ multilayer films assembled on slides in MB solutions with different ionic strengths. The loading of MB into LBL films reached adsorption equilibrium after 20 min for all different ionic strengths. The results showed that increasing the salt concentration affected the loading of MB solution. Therefore, the increase in NaCl concentration decreased the loading of MB. This could be related to the salting-out effect of the high salt concentration. Due to the high salt concentration, ionic bonds between two polymeric layers were disrupted. Thus, the layered structure was loosened, and layer separation occurred.

Figure 4B shows the effects of different pH values on the loading of MB into (Chi-g-PEI/PAA)_10_-multilayer-film-coated slides. PAA is a weak polyelectrolyte, and the functional groups of PAA chains are carboxyl (–COOH) ones. The increase in pH dissociated the functional groups on the polymer chain. With pH below 4.5, the functional groups on the PAA chains were predominantly undissociated, because the pKa value of PAA is 4.5 [32]. The pH value of the solutions affected the charge density of the PAA chains. It is known that higher pH values affect the carboxylic acid groups of PAA chains, which may change into negatively charged carboxylate groups [33,34]. This may have caused electrostatic attractive forces between methylene blue and the carboxylate groups. So, at high pH values, methylene blue molecules could easily be absorbed by PAA chains. The results showed that faster and higher loading of MB into film layers was allowed to happen at higher pH values. In this study, the highest loading rate of methylene blue was obtained at pH 9.0. For this reason, at high pH values, almost all the carboxyl groups of polyacrylic acid chains were converted to carboxylate groups. During the release process, the pH values of the immersion solutions were all above the pKa of the polyacrylic acid in the films. Therefore, the degrees of ionization of PAA were almost unchanged at pH 5.0, 7.0, and 9.0. After 6 min at pH 5, the MB amount loaded into film layers reached the saturation point. The same behaviors were observed after 10 min at pH 7.0 and after 15 min at pH 9.0.

### 3.3. Release Studies of Methylene Blue

#### 3.3.1. Effect of pH Value on Release

**Figure 5:**
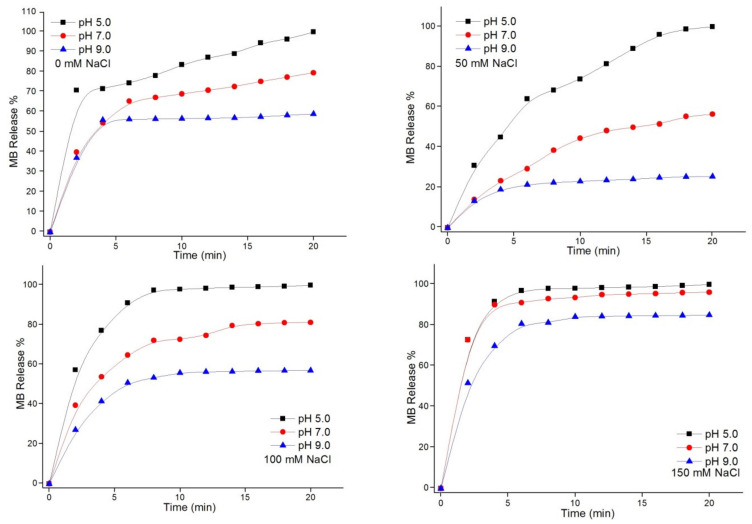
pH effects on release of MB from (Chi-g-PEI/PAA)_10_ multilayer films. MB was released in different salt solutions (0, 50, 100, and 150 mM NaCl) at pH 5.0, 7.0, and 9.0.

The effects of pH on the release rate of MB-loaded (Chi-g-PEI/PAA)_10_ films were evaluated at different NaCl concentrations, as shown in Figure 5. When the pH value decreased, a large amount of H^+^ in the solution penetrated into the films, and carboxylate groups (COO^−^) were protonated on the PAA chains. Thus, the MB molecule no longer interacted with the carboxylic acid groups (COOH) on the PAA chains at low pH values due to its positively charged structure [35]. This caused burst release, and it is not a desirable situation for sustained drug delivery. As a result, the electrostatic attractive forces between the carboxylic acid groups (COOH) of PAA and MB were strong at high pH values. In addition, the release rate was slow under basic conditions. The release rate of MB was increased with the decrease in the pH of the solution at all different salt concentrations. Additionally, there were no significant differences in the pH changes in 150 mM salt solution.

#### 3.3.2. Ionic-Strength Effect on Release of MB

**Figure 6:**
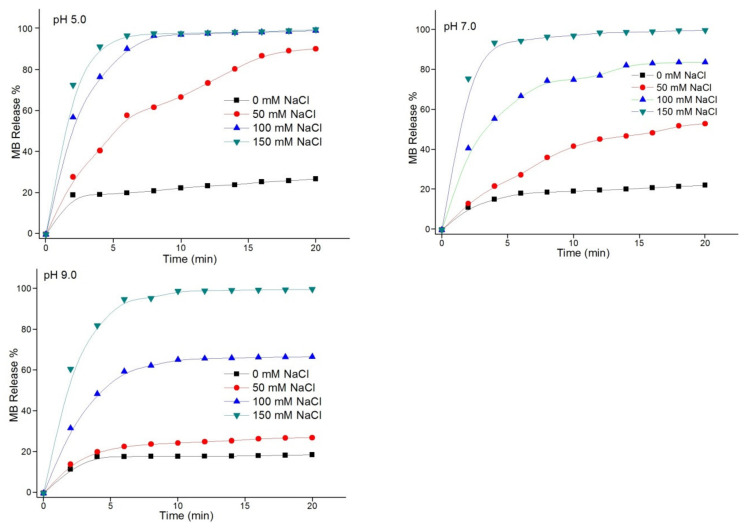
Ionic-strength effects on the release of MB from (Chi-g-PEI/PAA)_10_ multilayer films. MB was released in different salt solutions (0, 50, 100, and 150 mM NaCl) at pH 5.0, 7.0, and 9.0.

The effects of the salt concentration on the MB release rate from (Chi-g-PEI/PAA)_10_ multilayer films were investigated. MB-loaded (Chi-g-PEI/PAA)_10_ films were immersed in solutions at different NaCl concentrations (0 mM, 50 mM, 100 mM, and 150 mM) and pH values (5.0, 7.0, and 9.0). As shown in Figure 6, increasing the salt concentration also rapidly increased the release rate of MB, which showed an ionic-strength-controlled release behavior from (Chi-g-PEI/PAA)_10_ multilayer films. As of a result of the electrostatic screening process, an increase in ionic strength led to the loosening of the film structure, thereby resulting in enhanced permeability and quick MB-release process from the films. Consequently, these results indicated that the MB release rate from films could be adjusted by modifying the ionic solution strength.

### 3.4. Reversibility of MB Loading into and Release from (Chi-g-PEI/PAA)_10_ Films

**Figure 7:**
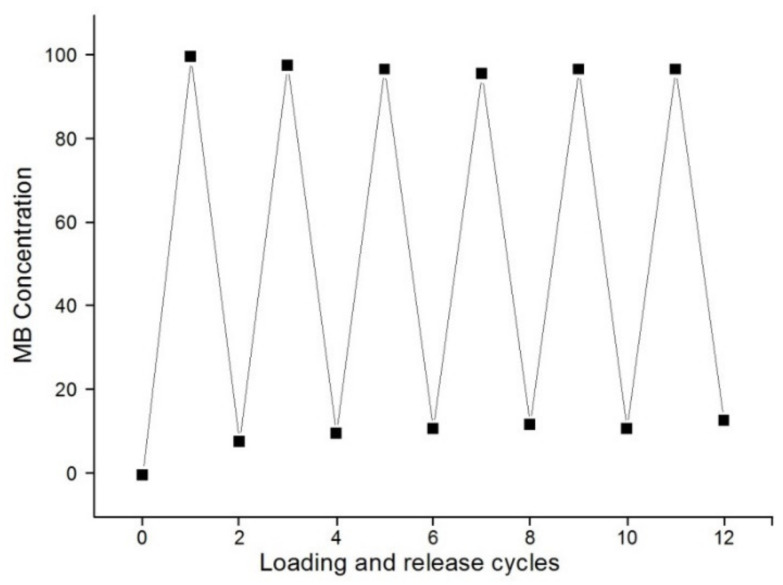
Cyclic absorbance features of (Chi-g-PEI/PAA)_10_ multilayer films at 668 nm.

The reversibility of MB loading into and release from (Chi-g-PEI/PAA)_10_ films was investigated. LBL-multilayer-film-assembled slides were dipped into 0.3 mg/mL MB solutions (0 mM NaCl) at pH 7.0 for 15 min. Then, the LBL-MB-loaded films were dipped in 150 mM NaCl solution at pH 7.0. This procedure was repeated for the desired number of times. As shown in Figure 7, the fluctuation in the MB amount was regular during the successive loading into and release from (Chi-g-PEI/PAA)_10_ films. According to the results, no reasonable changes were observed after five times, and the film layers could reversibly load and release drug molecules.

## 4. Conclusions

In this work, PEI-grafted chitosan was prepared and used for the formation of LBL films for drug-delivery applications. MB was used as a model drug to study drug delivery using Chi-g-PEI/PAA films. Both the ionic strength and pH value of the MB solution significantly affected MB loading into LBL films. The loading rate was decreased with the increase in ionic strength, further increasing as the pH was increased. Concerning the release process, the rate of release could be controlled via both the ionic strength and the pH value of the immersion solution. The acceleration of the release of MB from LBL films could be tailored via the administration of both high ionic strength and low pH. In addition, (Chi-g-PEI/PAA)_10_ multilayer films exhibited great reversibility in terms of the loading and release cycle of MB. Consequently, the release rate could be adjusted by adjusting pH and ionic strength. These results showed that (Chi-g-PEI/PAA)_10_ LBL multilayer films can be useful for the development of drug-delivery systems and other release applications.

## Data Availability

Not applicable.
